# Zinc Biofortification of Rice in China: A Simulation of Zinc Intake with Different Dietary Patterns

**DOI:** 10.3390/nu4060517

**Published:** 2012-06-14

**Authors:** Yu Qin, Alida Melse-Boonstra, Baojun Yuan, Xiaoqun Pan, Yue Dai, Minghao Zhou, Rita Wegmueller, Jinkou Zhao, Frans J. Kok, Zumin Shi

**Affiliations:** 1 Department of Chronic Disease Control and Prevention, Jiangsu Province Centre for Disease Control and Prevention, Nanjing 210009, China; Email: yu.qin@wur.nl (Y.Q.); yuanbj@jscdc.cn (B.Y.); pxq2411@yahoo.com.cn (X.P.); daiy@jscdc.cn (Y.D.); zmh@jscdc.cn (M.Z.); button.zhao@gmail.com (J.Z.); zumin.shi@gmail.com (Z.S.); 2 Division of Human Nutrition, Wageningen University, P.O. Box 8129, 6700 EV Wageningen, The Netherlands; Email: frans.kok@wur.nl; 3 Laboratory for Human Nutrition, ETH Zurich, CH-8092 Zürich, Switzerland; Email: rita.wegmueller@ilw.agrl.ethz.ch; 4 Discipline of Medicine, University of Adelaide, North Terrace Adelaide SA 5005, Australia

**Keywords:** zinc, biofortification, dietary intake, dietary pattern, simulation, China

## Abstract

A cross-sectional survey of 2819 adults aged 20 years and above was undertaken in 2002 in Jiangsu Province. Zinc intake was assessed using a consecutive 3-day 24-h dietary recall method. Insufficient and excess intake was determined according to the Chinese Dietary Recommended Intakes. Four distinct dietary patterns were identified namely “traditional”, “macho”, “sweet tooth”, and “healthy”. Intake of zinc from biofortified rice was simulated at an intermediate zinc concentration (2.7 mg/100 g) and a high zinc concentration (3.8 mg/100 g) in rice. Average total zinc intake was 12.0 ± 3.7 mg/day, and insufficiency of zinc intake was present in 15.4%. Simulated zinc intake from biofortified rice with intermediate and high zinc concentration decreased the prevalence of low zinc intake to 6.5% and 4.4%, respectively. The effect was most pronounced in the “traditional” pattern, with only 0.7% of insufficiency of zinc intake remaining in the highest quartile of the pattern. Zinc intake was inversely associated with the “sweet tooth” pattern. Zinc biofortifed rice improves dietary zinc intake and lowers risk for insufficient zinc intake, especially for subjects with a more “traditional” food pattern, but less for subjects with a “sweet tooth” food pattern.

## 1. Introduction

Zinc deficiency is prevalent and affects nearly two billion people in the developing world, where mainly cereals are consumed by the population [1]. Zinc deficiency results in retarded growth [2], higher morbidity and mortality from infectious diseases in children [3,4], impaired pregnancy and infant outcome [5], and is also associated to several chronic diseases in adults, such as diabetes and malignancy [6]. Although there is still no accurate indicator for zinc deficiency, dietary zinc intake appears to adequately predict zinc status among adults [7]. 

In a previous study we have shown that daily dietary zinc intake in Jiangsu Province is low according to the Chinese Dietary Recommended Intakes, especially in children and adolescents [8]. Zinc content is highest in animal-source foods, relatively high in whole-grain cereals, and low in refined cereals and vegetables [9]. Rice is one of the most important staple foods and is an important source of minerals and trace elements in China. Cereal grains and vegetables together contribute 50%–70% to dietary zinc intake in the Chinese population [10], which is higher than the proportion (27%) in western countries [11]. One of the strategies to improve dietary zinc intake is to increase zinc content in staple crops through genetic or agronomic strategies, or through selective breeding, which is called biofortification. These techniques have the potential to combat zinc deficiency, although there is still no direct evidence to demonstrate this [12]. 

Simulation models using dietary intake data in a representative target population are a useful tool to evaluate food-based interventions, such as biofortification or fortification [13]. Such models have been used in establishing safe and potentially efficacious fortification for some micronutrients, like folic acid, and calcium [13,14,15,16]. However, this way of analysis is still limited, and only a few of such studies have focused on zinc, one from Bangladesh [17] and one from Mexico [18]. 

In order to identify individuals at risk for nutrition related complaints, dietary patterns rather than the traditional single food item approach has been developed using principal factor analysis [19,20,21,22,23]. Such analyses are useful to evaluate associations between dietary patterns and risk of diseases. Such dietary patterns have been defined in relation to anemia and obesity for the population of Jiangsu Province, China, based on data from a sub-group of a national dietary intake survey [23,24]. In the present study we aimed to describe the effect of simulated zinc intake from biofortified rice on dietary zinc intake and to compare the effect of biofortification between different dietary patterns among adults in the Province.

## 2. Materials and Methods

### 2.1. Sample

The study was conducted in Jiangsu Province using a multistage cluster sampling method, as described before [8,24], which was part of the 2002 National representative cross-sectional survey on nutrition and health. Six counties and two prefectures were included, which represented a geographically and economically diverse population for Jiangsu Province. From each of the six areas, three streets/towns were randomly selected. In each street/town, two villages/neighbourhoods were further randomly selected. In each village/neighborhood, thirty households were randomly selected. All members in the households were invited to take part in the study and written consent was obtained from all the participants with a response rate of 90.8%. Altogether, 2819 adults aged 20 years and older with complete data were included in our analysis. The study was approved by the Human Investigation Review Committee at the National Institute for Nutrition and Food Safety, Chinese Center for Disease Control and Prevention.

### 2.2. Dietary Intake Measurement

Trained interviewers from the local Center for Disease Control and Prevention visited subjects in their homes to collect the information on food intake using a 24-h dietary recall method on three consecutive days, including two weekdays and one weekend day. Energy and nutrient intake was calculated using the data of the dietary recall in conjunction with the China Food Composition Table which was updated in 2002 [25]. 

### 2.3. Determination of Dietary Pattern

Dietary patterns were identified with data collected by a food frequency questionnaire (FFQ), using standard principal component analysis as described before for this population [24]. The FFQ was validated, and used to collect dietary information over the previous year [26]. The FFQ included a series of detailed questions regarding the usual frequency and quantity of intake of thirty-three foods and beverages. This was further merged into twenty-five food items in the analysis because of the low intake of some food items. Portion size for each food was established by reference to food models. Subjects were asked to recall the frequency of consumption of individual food items (number of times per day, per week, per month, per year) and the estimated portion size, using local weight units (1 liang = 50 g) or natural units (cups). Intakes of foods were converted into g/week for data analysis. Use of vitamin and mineral supplements was included in the questionnaire, but because these were very seldom used in the area, they were not included in this analysis. 

For identification of the dietary patterns, factor loading for each food item was calculated, which is equivalent to a simple correlation between the food item and the factor (Table 1). Higher loadings (absolute value) indicate that the food shares more variance with that factor. The sign of the loading determines the direction of the relationship of each food to the factor. Food groups with absolute values of less than 0.20 are excluded from the table for simplicity. Only one food item (cheese) is missing owing to low factor loading. Wheat flour includes noodles and steamed dumplings, and beverages include soft drinks, coffee and tea.

**Table 1:** Factor loading for four dietary patterns among adults.

Factor 1: “Traditional” pattern			Factor 2: “Macho” pattern			Factor 3: “Sweet tooth” pattern			Factor 4: “Healthy” pattern	
Food or food group	Factor loading		Food or food group	Factor loading		Food or food group	Factor loading		Food or food group	Factor loading
Rice	0.81		Poultry	0.56		Cake	0.60		Whole grains	0.54
Fresh vegetables	0.57		Beer	0.53		Juice	0.58		Fruits	0.49
Pork	0.37		Beef, lamb	0.46		Beverage	0.48		Pickled vegetables	0.46
Fish	0.21		Deep-fried products	0.45		Milk	0.48		Tofu	0.44
Root vegetable	−0.32		Pork	0.44		Yoghurt	0.44		Fresh vegetables	0.37
Wheat flour	−0.78		Liver	0.43		Beef, lamb	0.30		Root vegetable	0.34
			Alcohol	0.43		Nut	0.26		Milk	0.31
			Eggs	0.38		Poultry	0.25		Eggs	0.30
			Fish	0.26		Fruits	0.23		Fish	0.24
			Nuts	0.23		Pickled vegetables	−0.20		Wheat flour	0.23
			Fruits	0.22		Alcohol	−0.27		Milk powder	0.22
			Tofu	0.22					Beer	−0.21

Factor loadings are equivalent to a simple correlation between the food items and the factor. Higher loadings (absolute value) indicate that the food shares more variance with that factor. The sign of the loading determines the direction of the relationship of each food to the factor. Food groups with absolute values of less than 0.20 are excluded from the table for simplicity. Only one food item (cheese) is missing owing to low factor loading. Wheat flour includes noodles and steamed dumplings. Beverages include soft drinks, coffee and tea.

Four different patterns were defined: (1) the “traditional” pattern, characterized by high intakes of rice and fresh vegetables and low intake of wheat flour; (2) the “macho” pattern, characterized by intake of animal foods and alcohol, *i.e.*, foods commonly eaten by men; (3) the “sweet tooth” pattern, characterized by intake of cake, milk, yoghurt and beverages; and (4) the “healthy” pattern, characterized by intake of whole grain products, fruits, root vegetables, and fresh and pickled vegetables. The four patterns explained 30.5% of the variance in intake (10.6%, 8.6%, 5.9% and 5.4% for “traditional”, “macho”, “sweet tooth”, and “healthy” patterns, respectively) (Table 1). Scores for each pattern were calculated as the sum of the products of the factor loading coefficient and the standardized weekly intake of each food associated with that pattern. Only foods with factor loadings of more than 0.20 or less than −0.20 were included in the calculation of pattern scores because these items represent the foods most strongly related to the identified factor. 

### 2.4. Socio-Economic Status

Socio-economic status (SES) was assessed by the question “What was your family’s income per person in 2001?” Low SES was defined as an income of less than 1999 Yuan, “medium” as 2000–4999 Yuan and “high” as more than 5000 Yuan.

### 2.5. Simulated Biofortification of Rice

The mean zinc concentration in normal rice was 1.7 mg/100 g (raw polished rice) based on the dietary China Food Composition Table (2002) [25]. Ma *et al*. [27] have reported that phytic acid and zinc concentration in four types of raw rice consumed in China ranged from 55 to 183 mg phytic acid/100 g (average 115 mg/100 g) and 1.09 to 1.76 mg zinc/100 mg (average 1.41 mg/100 g), respectively. The phytate/zinc molar ratio ranged from 3.07 to 11.27 (average 8.4). A specific variety with a zinc concentration of 3.8 mg/100 g and 290 mg/100 g phytate (phytate/zinc molar ratio 7.6) in raw polished rice (Qinai-3-hun, a Chinese variety) has recently been produced in a greenhouse by breeding under controlled conditions at Wageningen University, the Netherlands (unpublished results). The limit of zinc in biofortified rice appears to be about 10 mg/100 g, and zinc concentrations of 3.5 mg/100 g have previously been reported in paddy-grown rice [28]. In our study, we simulated two levels of zinc in biofortified rice, namely a high level (3.8 mg/100 g) and an intermediate level (2.7 mg/100 g). 

### 2.6. Statistical Analysis

Variables are presented as mean ± standard deviations (SD) for numeric variables or as percentages for categorical variables. Insufficiency of zinc intake was determined as below 2/3 of the recommended nutrient intake (RNI) with age- and gender-specific values according to the Chinese DRIs, which is 15 mg/day for males (18–49 years), and 11.5 mg/day for males (>49 years) and for females (>19 years) [29]. Furthermore, excessive zinc intake was defined as zinc intake higher than the cut-off of the tolerable upper intake level, which is 45 mg/day for males (20–50 years) and 37 mg/day for females (>20 years) and for males (>50 years) [29]. Absorbed zinc was estimated according to IZiNCG guidelines, with zinc absorption fractions of 26% in men and 34% in women consuming a mixed diet. Consequently, absorbed zinc inadequacy was defined as absorbed zinc levels lower than 2.69 mg/day for men and 1.86 mg/day for women [9]. Dietary pattern scores were categorized to quartiles from Q1 (the lowest) to Q4 (the highest).This implies that the highest quartile consists of subjects that best represented each of the dietary patterns. Paired *t*-test was applied to analyze differences in zinc intake from normal rice as compared to simulated zinc intake at the two levels of biofortification. ANOVA and chi-squared test was used to determine group differences for continuous and qualitative variables, respectively. Linear regression was performed for zinc intake over the quartiles in dietary pattern scores, adjusted by household, age, gender, region and area of residence, SES, education and energy intake. All analyses were performed using SPSS 19.0 (SPSS Inc., Chicago, IL, USA). Statistical significance was set at α = 0.05.

## 3. Results

The study included 2819 participants, with 1297 males and 1522 females. There were no differences in age, residence (urban/rural), region (north/south), socio-economic status and education level between genders. The average rice intake was 250.1 ± 145.8 g/day, with no difference between males and females. About 5% of the participants reported not to eat rice. 

Table 2 shows the characteristics in the highest quartile of each of the four dietary patterns. There were no gender differences in each of the dietary patterns. The “traditional” pattern was associated positively with age, whereas the “macho” and “sweet tooth” patterns were inversely associated with age. For all patterns, the rural residents were represented with a higher proportion. However, the difference in the proportion between rural and urban residents was larger in the “traditional” pattern than in the other patterns. The “traditional”, “macho” and “sweet tooth” patterns were more frequently represented in those with high SES. Compared to the South, subjects from the North reflected more the “healthy pattern” and less any of the other patterns. The “traditional” and “healthy” patterns were more represented in those with low education level, whereas the “macho” and “sweet tooth” patterns were more represented in the medium and high education categories. 

**Table 2:** Sample characteristics in the highest quartile of dietary patterns.

		All (%)	Traditional	Macho	Sweet tooth	Healthy
Gender	Male	1297 (46.0)	46.9	44.5	45.9	48.5
	Female	1522 (54.0)	53.1	55.5	54.1	51.5
Age group	20–29	307 (10.9)	8.7	13.7	18.8	11.3
	30–39	589 (20.9)	16.4	25.2	22.0	21.1
	40–49	610 (21.6)	23.1	25.5	19.3	23.3
	50–59	527 (18.7)	26.3	16.8	16.6	17.0
	60+	786 (27.9)	25.4	18.7	23.3	27.3
Residence	Urban	703 (24.9)	18.3	39.8	49.6	38.2
	Rural	2116 (75.1)	81.7	60.2	50.4	61.8
Region	South	1486 (52.7)	82.3	54.8	68.0	41.9
	North	1333 (47.3)	17.7	45.2	32.0	58.1
SES	Low	911 (32.2)	10.9	23.2	14.4	39.8
	Medium	899 (31.9)	41.7	34.6	27.4	27.2
	High	984 (34.9)	47.4	42.2	58.1	33.0
Education	Primary	1343 (47.6)	45.9	29.2	28.3	41.4
	Junior school	1024 (36.3)	36.4	47.4	39.1	35.8
	High school	451 (16.1)	17.7	23.4	32.6	22.8

SES, socio-economic status. Data are presented as percentages of the total number of subjects in the fourth quartile of each of the dietary patterns.

Mean zinc intake in the population was 12.0 ± 3.7 mg/day and was not different for males and females (12.1 ± 3.7 mg/day *vs*. 12.0 ± 3.7 mg/day, respectively). The average zinc intake from normal rice was 4.1 mg/day, and represented 34.3% of total zinc intake. The prevalence of insufficiency of zinc intake was 15.4%. The estimated amount of absorbed zinc was 3.6 mg/day, with 16.2% of the population showing absorbed zinc inadequacy. None of the subjects consumed zinc at higher intakes than the cut-off of the tolerable upper intake level (Table 3). 

**Table 3:** Estimated zinc intake from a diet with normal rice and biofortified rice.

	Normal rice	Biofortified rice	
		Intermediate level	High level
Zinc concentration in rice (mg/100 g)	1.7	2.7	3.8
Zinc intake from rice (mg/day)	4.1 ± 2.7	6.5 ± 4.3 ^†^	9.2 ± 6.1 ^†^
% of total zinc intake	34.3	45.4	53.9
% of insufficiency of zinc intake	15.4	6.5 ^‡^	4.4 ^‡^
Estimated absorbed zinc intake	3.6 ± 1.2	4.4 ± 1.4 ^†^	5.2 ± 1.7 ^†^
% of absorbed zinc inadequacy	16.2	7.3 ^‡^	5.2 ^‡^

Insufficiency of zinc intake was determined as below 2/3 of the recommended nutrient intake (RNI) with age- and gender-specific values according to the Chinese DRIs. Absorbed zinc intake was estimated as 26% for men and 34% for women, according to IZiNCG recommendations for populations with a mixed diet. Absorbed zinc inadequacy was defined as absorbed zinc intake lower than 2.69 mg/day for men and 1.86 mg/day for women. ^†^ Paired t-test in comparison with normal rice, *P* < 0.001. ^‡^ Chi-square test compared with normal rice, *P* < 0.001.

Simulated zinc intake at the two levels of zinc from biofortified rice was significantly higher than that from normal rice, with 6.5 and 9.2 mg/day for the intermediate and high zinc level (*P* < 0.001), respectively. The prevalence of insufficiency of zinc intake decreased to 6.5% and 4.4% for the intermediate and high level of biofortification (*P* < 0.001), respectively. Estimated absorbed zinc intake improved accordingly, with 7.3% and 5.2% of subjects remaining with absorbable zinc inadequacy, respectively. One out of 2819 (0.03%) participants had an excessive intake of zinc with a diet including the intermediate biofortification level, and four out of 2819 (0.1%) with the high biofortification level (Table 3). 

Energy intake decreased with the “sweet tooth” pattern, and increased with the other three patterns (Table 4). Daily rice intake was highest in the highest quartile of the “traditional” pattern, and decreased with the “sweet tooth” and “healthy” patterns. Average zinc intake increased with the “traditional” and decreased with the “sweet tooth” pattern. The prevalence of insufficiency of zinc intake was lower with the “traditional” and “macho” patterns, and higher with the “sweet tooth” pattern. No association was found between the “healthy” pattern and prevalence of insufficiency of zinc intake (Table 4). 

When replacing normal rice with biofortified rice, the largest increases in dietary zinc intake were seen in the highest quartile of the “traditional” pattern (+3.5 mg/day and +7.3 mg/day for biofortification at intermediate and high level, respectively). The prevalence of insufficiency of zinc intake after biofortification decreased for all of the dietary patterns. For the “traditional” pattern, the prevalence of insufficiency of zinc intake decreased to only 0.7% in the highest quartile of the “traditional” pattern after simulating zinc intake with biofortified rice at the highest zinc level. 

**Table 4:** Dietary zinc intake (mg/day) and prevalence of insufficiency of dietary zinc intake (%) with normal rice and biofortified rice in each of the dietary patterns.

Dietary patterns		Energy (1000 kcal/day)	Rice (g/day)	Normal rice(1.7 mg/100 g)			Biofortified rice(2.7 mg/100 g)			Biofortified rice(3.8 mg/100 g)	
				Zinc intake, mg/day	Insufficiency %		Zinc intake, mg/day	Insufficiency %		Zinc intake, mg/day	Insufficiency %
Traditional	Q1	2.5 (0.7)	111.2 (97.9)	12.1 (4.0)	18.4		13.1 (4.2)	11.5		14.3 (4.7)	9.3
	Q2	2.2 (0.6)	215.1 (109.2)	11.0 (3.6)	23.3		13.2 (4.1)	10.9		15.5 (5.2)	7.1
	Q3	2.2 (0.6)	309.0 (104.0)	11.9 (3.3)	11.3		15.0 (3.5)	2.1		18.4 (4.1)	0.4
	Q4	2.5 (0.7)	367.0 (122.2)	13.1 (3.4)	8.4		16.6 (4.0)	1.6		20.4 (4.7)	0.7
*P* for trend		<0.001	<0.001	0.003	<0.001		<0.001	<0.001		<0.001	<0.001
Macho	Q1	2.3 (0.7)	247.6 (12.4)	11.6 (3.7)	18.8		14.0 (4.1)	7.6		16.7 (5.1)	5.1
	Q2	2.3 (0.6)	152.9 (143.6)	11.7 (3.4)	14.4		14.2 (3.9)	6.7		16.9 (5.0)	4.0
	Q3	2.3 (0.7)	255.2 (138.0)	12.1 (3.8)	16.3		14.6 (4.3)	7.5		17.3 (5.4)	5.7
	Q4	2.5 (0.7)	244.6 (137.8)	12.7 (3.9)	12.0		15.1 (4.4)	4.3		17.7 (5.5)	2.9
*P* for trend		<0.001	0.51	0.401	0.002		0.968	0.04		0.565	0.05
Sweet tooth	Q1	2.7 (0.7)	258.1 (166.8)	13.5 (4.0)	6.8		15.9 (4.4)	2.8		18.7 (5.4)	2.1
	Q2	2.4 (0.7)	261.8 (158.2)	12.0 (3.7)	15.5		14.5 (4.1)	6.4		17.3 (5.2)	4.3
	Q3	2.2 (0.6)	255.3 (138.4)	11.6 (3.3)	17.8		14.0 (3.9)	7.1		16.7 (5.0)	4.5
	Q4	2.1 (0.6)	225.0 (111.0)	11.0 (3.4)	21.4		13.3 (3.9)	9.8		15.9 (5.1)	6.7
*P* for trend		<0.001	<0.001	0.037	<0.001		<0.001	<0.001		<0.001	0.001
Healthy	Q1	2.3 (0.7)	290.2 (131.7)	11.9 (3.7)	16.9		14.5 (4.2)	6.6		17.4 (5.1)	4.9
	Q2	2.3 (0.6)	270.1 (151.5)	12.0 (3.5)	14.8		14.5 (4.0)	6.0		17.2 (5.1)	3.7
	Q3	2.4 (0.7)	226.0 (148.5)	11.8 (3.6)	16.2		14.1 (4.2)	8.3		16.7 (5.5)	5.5
	Q4	2.5 (0.7)	213.9 (137.6)	12.5 (4.0)	13.6		14.7 (4.4)	5.2		17.3 (5.3)	3.4
*P* for trend		<0.001	<0.001	0.821	0.65		0.021	0.13		0.001	0.16

Data are presented by mean (SD) or percentage. Q1 is the lowest and Q4 the highest quartile of each dietary pattern. Insufficiency of zinc intake was determined as below 2/3 of the recommended nutrient intake (RNI) with age- and gender- specific values according to the Chinese DRIs and was analyzed by chi-square test over dietary patterns. Energy and rice intake was analyzed by ANOVA. Dietary zinc intake over dietary patterns was analyzed by linear regression, adjusted for household, age, gender, resident, region, SES, education and energy intake.

## 4. Discussion

In our study, simulated zinc intake from biofortified rice at two levels of zinc resulted in an increase in total zinc intake and a decrease in the prevalence of insufficient zinc intake in an adult population. Rice was the most important staple food in our study population, with 95% of subjects eating rice daily. Only 0.1% of the participants would have excessive zinc intake with rice biofortified at the highest level (3.8 mg/100 g). 

There are some limitations in the present study that should be mentioned. Firstly, day-to-day variation is the main random error of dietary recall, which we have minimized by using 3-day 24 h dietary recalls, including two weekdays and one weekend day, in addition to a validated FFQ conducted by well-trained interviewers. Nevertheless, over- or underreporting may have occurred resulting in misclassification, thereby weakening the associations under study. Secondly, since data on dietary phytate was not available, we could not calculate the exact bioavailability of zinc for each individual. Instead, we have assumed that the mixed diets of people in Jiangsu Province are inhibitory for zinc absorption at the intermediate level based on the suggestions by IZiNCG [9]. When estimating intake of absorbable zinc, the adequacy of absorbed zinc intake was very similar to insufficiency of total zinc intake. Moreover, we used a cut-off of 2/3 Chinese RNI for assessment of insufficient zinc intake. This cut-off corresponds well with the IZiNCG EAR at the age of 18–49 years [9]. Thirdly, a common limitation for factor analysis is arbitrary decisions in determining the number of factors to retain and in labeling food patterns [20,30]. Also, a causal relationship between dietary pattern and insufficient zinc intake cannot be derived from this cross-sectional study. 

The use of simulated zinc intake in the design of programs for micronutrient fortification or biofortificaiton is limited. Consistent with our report, a Mexican study showed that simulated biofortification of maize and beans with additional amounts of zinc resulted in a significantly decreased prevalence of inadequate absorbed zinc intake [18]. Subjects in our study consumed 12 mg/day of total dietary zinc and 3.6 mg/day of estimated absorbed zinc, and the prevalence of insufficient zinc intake and absorbed zinc inadequacy was around 15%–16%. Mexican women, however, only consumed 1.68 mg/day of absorbable zinc, and the prevalence of absorbed zinc inadequacy was between 40% and 50% [18]. Like our study, Arsenault *et al*. [17] also reported the effect of simulated increases in the zinc content of rice on improvements in total dietary zinc adequacy in rural Bangladeshi children and women In the women, mean intakes of total zinc and absorbed zinc after simulation were 5.5 and 1.3 mg/day, with a prevalence of absorbed zinc inadequacy of 76%–99.8%, compared with 100% at baseline [17]. The discrepancies between studies show that the effect of biofortification depends on the magnitude of zinc deficiency in a specific population. Moreover, differences in the simulated amount of zinc from biofortification may also result in different effects. In the Bangladeshi population, an additional 0.8 mg of zinc/100 g of rice (raw weight, with unknown baseline zinc concentration) was added [17], whereas we added 1.0 mg/100 g and 2.1 mg/100 g in our simulations. The modest increment of zinc in rice may have contributed to the remaining high prevalence of absorbed zinc inadequacy in the Bangladeshi study. 

Biofortification of rice provides a potential approach to improve zinc intake in populations with rice as a staple food, because it does not require a change in the choice of staple foods. Since the phytate/zinc molar ratio is lower than 15 in rice in China [27], it can be expected that zinc from rice is well absorbed. For this study, we have used two levels of biofortification: 2.7 mg/100 g and 3.8 mg/100 g. The latter level was derived from biofortified rice that has been grown at Wageningen University on Zn-fortified solution culture medium in a greenhouse (unpublished results). It still needs to be shown whether this high level of biofortification can also be reached under field conditions, although there is some evidence [28]. Since a biofortified staple food is more difficult to adjust to the specific needs of populations than commercial fortified foods [12], it is very important to set efficacious target breeding levels. According to our simulations, the largest reduction in prevalence of zinc intake insufficiency was achieved with the intermediate level of zinc. Therefore, a level of 2.7 mg/100 g may be set as preliminary goal for the first phase of development of biofortified rice, which is very close to the target zinc content in rice (2.8 mg/100 g) that has recently been recommended by HarvestPlus [28]. 

With respect to dietary patterns, the positive effect of biofortification on dietary zinc was much more significant in the highest quartile of the “traditional” pattern, which can be attributed to the higher intake of rice in this population segment. In contrast, there was an inverse trend between zinc intake and the “sweet tooth” pattern. High intake of beverages, milk and cake may contribute to poor zinc intake. Zinc concentrations have been reported to be low in distilled drinks and milk [9,31]. Moreover, calcium is abundant in milk, which may have an inhibitory effect on zinc absorption [32]. 

## 5. Conclusion

In conclusion, we found that zinc biofortifed rice improves dietary zinc intake and lowers the risk for insufficient zinc intake, especially for subjects with a “traditional” dietary pattern among adults in Jiangsu Province, where rice is the main staple food. The positive effect is attenuated with a “sweet tooth” dietary pattern due to the lower intake of rice. Biofortification of rice offers a promising opportunity to reduce zinc deficiency in China, in addition to other diet-based strategies such as dietary diversification and health education. Further studies should focus on the breeding level of Zn that can actually be achieved in rice when grown under various field conditions in China, as well as its cost-effectiveness. Furthermore, the absorption of zinc from zinc biofortified rice should be assessed.
